# Hormone receptor status may impact the survival benefit of surgery in stage IV breast cancer: a population-based study

**DOI:** 10.18632/oncotarget.11235

**Published:** 2016-08-11

**Authors:** Yinuo Tan, Xiaofen Li, Haiyan Chen, Yeting Hu, Mengjie Jiang, Jianfei Fu, Ying Yuan, Kefeng Ding

**Affiliations:** ^1^ Department of Surgical Oncology, 2nd Hospital of Zhejiang University School of Medicine, Hangzhou, P.R. China; ^2^ Cancer Institute, Key Laboratory of Cancer Prevention and Intervention, China National Ministry of Education, Key Laboratory of Molecular Biology in Medical Sciences, Hangzhou, Zhejiang Province, China, and The Second Affiliated Hospital, Zhejiang University School of Medicine, Hangzhou, P.R. China; ^3^ Department of Medical Oncology, 2nd Hospital of Zhejiang University School of Medicine, Hangzhou, P.R. China; ^4^ Department of Oncology, Jinhua Central Hospital, Jinhua, P.R. China

**Keywords:** metastatic breast cancer, surgery, prognosis, SEER

## Abstract

**Introduction:**

The role of surgery in stage IV breast cancer is controversial. We used the Surveillance, Epidemiology, and End Results database to explore the impact of surgery on the survival of patients with stage IV breast cancer.

**Methods:**

In total, 10,441 eligible stage IV breast cancer patients from 2004 to 2008 were included. They were divided into four groups as follows: R0 group (patients who underwent primary site and distant metastatic site resection), primary site resection group, metastases resection group, and no resection group.

**Results:**

The four groups achieved a median survival time (MST) of 51, 43, 31 and 21 months, respectively, *P* < 0.001. The Cox proportional hazards model showed that the R0 group, primary resection group and metastases resection group had a good survival benefit, with hazard ratios of 0.558 (95% CI, 0.471-0.661), 0.566 (95% CI, 0.557-0.625) and 0.782 (95% CI, 0.693-0.883), respectively. In the hormone receptor (HR)-positive population, the R0 group (MST = 66 m, 5-year OS = 54.1%) gained an additional survival benefit compared with the primary resection group (MST = 52 m; 5-year OS = 44.9%; *P* < 0.001). The metastases resection group (MST = 38 m; 5-year OS = 31.7%) survived longer than the no resection group (MST = 28 m; 5-year OS = 22.0%; *P* < 0.001). In the HR-negative population, the R0 group and primary resection group had a similar survival (*P* = 0.691), and the metastases resection group had a similar outcome to that of the no resection group (*P* = 0.526).

**Conclusion:**

Patients who underwent surgery for stage IV breast cancer showed better overall survival than the no resection group. Cytoreductive surgery could provide a survival benefit in HR+ stage IV breast cancer; however, in the HR- population, extreme caution should be exercised when considering surgery.

## INTRODUCTION

Traditionally, metastatic breast cancer would be considered an incurable disease, in which the systemic approaches were recommended. However, it has been debated for years whether surgery could actually attain a survival benefit in metastatic breast cancer. Since 2002, several retrospective clinical studies have shown that primary tumor resection is correlated with significantly increased survival in patients with primary metastatic breast cancer[1];however, other studies have indicated that surgery in metastatic breast cancer does not translate into a significant survival benefit. Breast cancer has clinical and biological heterogeneity, and the major subtypes of breast cancer are classified by three markers: estrogen receptor (ER), progesterone receptor (PR), and human epidermal growth factor 2-neu (HER2). Many recent findings have indicated that hormone receptor (HR) positivity may correlate with a better outcome of the breast cancer patient. It has been rarely studied whether surgery for metastatic breast cancer with different hormone receptor status could achieve a different survival benefit. Thus, we conducted a retrospective population-based study to explore the survival benefit of surgery in stage IV breast cancer using the Surveillance, Epidemiology, and End Results (SEER) program data.

## RESULTS

### Clinical and pathological features

The clinical and pathological features of the study population (*n* = 10,441) are shown in Table 1. The median OS was 29.0 months. Both primary site and distant metastatic site resections were performed in 272 (2.61%) patients (R0 group), 4,025 patients (38.55%) underwent primary site resection only, 409 patients (3.92%) underwent metastases resection only, and 5,735 patients (54.93%) had no surgery. The characteristics of the HR+ and HR- populations are shown in Supplementary Table S1.

**Table 1:** Clinical and pathological features of the study population

Variance	No.(%) of patients					
	R0 resection (*n*= 272)	Primary resection(*n*= 4025)	Metastases resection(*n*= 409)	No resection(*n*= 5735)	Total(*n*= 10441)	*P* value
**Age**						<0.001
≤45years	56 (20.6)	713 (17.7)	67 (16.4)	664 (11.6)	1500 (14.4)	
>45years	216 (79.4)	3312 (82.3)	342 (83.6)	5071 (88.4)	8941 (85.6)	
**Race**						0.025
White	222 (81.6)	3098 (77.0)	322 (78.7)	4363 (76.1)	8005 (76.7)	
Black	33 (12.1)	622 (15.5)	64 (15.6)	989 (17.2)	1708 (16.4)	
Other	15 (5.5)	296 (7.4)	22 (5.4)	359 (6.3)	692 (6.6)	
Unknown	2 (0.7)	9 (0.2)	1 (0.2)	24 (0.4)	36 (0.3)	
**Grade**						<0.001
Well	19 (7.0)	262 (6.5)	19 (4.6)	284 (5.0)	584 (5.6)	
Moderate	98 (36.0)	1291 (32.1)	87 (21.3)	1460 (25.5)	2936 (28.1)	
Poor	121 (44.5)	2146 (53.3)	96 (23.5)	1867 (32.6)	4230 (40.5)	
Unknown	34 (12.5)	326 (8.1)	207 (50.6)	2124 (37.0)	2691 (25.8)	
**Stage T**						<0.001
T0	5 (1.8)	4 (0.1)	32 (7.8)	165 (2.9)	206 (2.0)	
T1	55 (20.2)	611 (15.2)	44 (10.8)	436 (7.6)	1146 (11.0)	
T2	84 (30.9)	1382 (34.3)	69 (16.9)	950 (16.6)	2485 (23.8)	
T3	35 (12.9)	610 (15.2)	21 (5.1)	474 (8.3)	1140 (10.9)	
T4	71 (26.1)	1217 (30.2)	96 (23.5)	1984 (34.6)	3368 (32.3)	
Tx	22 (8.1)	201 (5.0)	147 (35.9)	1726 (30.1)	2096 (20.1)	
**Stage N**						<0.001
0	56 (20.6)	787 (19.6)	100 (24.4)	1271 (22.2)	2214 (21.2)	
1	80 (29.4)	1308 (32.5)	116 (28.4)	1956 (34.1)	3460 (33.1)	
2	51 (18.8)	745 (18.5)	21 (5.1)	347 (6.1)	1164 (11.1)	
3	58 (21.3)	877 (21.8)	30 (7.3)	502 (8.8)	1467 (14.1)	
NX	27 (9.9)	307 (7.7)	142 (34.7)	1659 (28.9)	2136 (20.5)	
**Radiation**						<0.001
Done	135 (49.6)	1703 (42.3)	163 (39.9)	1685 (29.4)	3686 (35.5)	
None	127 (46.7)	2216 (55.1)	219 (53.5)	3979 (69.4)	6541 (62.6)	
Unknown	10 (3.7)	106 (2.6)	27 (6.6)	71 (1.2)	214 (2.0)	
**ER**						<0.001
Positive	180 (66.2)	2589 (64.3)	248 (60.6)	3403 (59.3)	6420 (61.5)	
Negative	75 (27.6)	1156 (28.7)	78 (19.1)	1241 (21.6)	2550 (24.4)	
Unknown	17 (6.3)	280 (7.0)	83 (20.3)	1091 (19.0)	1471 (14.1)	
**PR**						<0.001
Positive	148 (54.4)	1990 (49.4)	172 (42.1)	2574 (44.9)	4884 (46.8)	
Negative	104 (38.2)	1706 (42.4)	144 (35.2)	1953 (34.1)	3907 (37.4)	
Unknown	20 (7.4)	329 (8.2)	93 (22.7)	1208 (21.1)	1650 (15.8)	
**HR**						<0.001
HR+	184 (67.6)	2646 (65.7)	252 (61.6)	3472 (60.5)	6554 (62.8)	
HR-	70 (25.7)	1095 (27.2)	74 (18.1)	1162 (20.3)	2401 (23.0)	
Unknown	18 (6.6)	284 (7.1)	83 (20.3)	1101 (19.2)	1486 (14.2)	
**Metastatic site**						<0.001
Distant Lymph node	48 (17.6)	334 (8.3)	22 (5.4)	192 (3.3)	596 (5.7)	
Designated organs^a^	94 (34.6)	1709 (42.5)	167 (40.8)	2393 (41.7)	4363 (41.8)	
Other organs	78 (28.7)	1541 (38.3)	130 (31.8)	2164 (37.7)	3913 (37.5)	
Multiple^b^	44 (16.2)	373 (9.3)	81 (19.8)	913 (15.9)	1411 (13.5)	
Unknown	8 (2.9)	68 (1.7)	9 (2.2)	73 (1.3)	158 (1.5)	

HR+ was defined as ER+ or PR+. HR- was defined as both ER- and PR-.

a Designated organs, metastasis in the following organs: adrenal (suprarenal) gland, bone, other than the adjacent rib, contralateral (opposite) breast, lung, ovary, satellite nodule(s) in skin other than the primary breast.

b Multiple mean metastases in at least two of the above sites.

### Influence of surgery on the overall outcome

**Figure 1:**
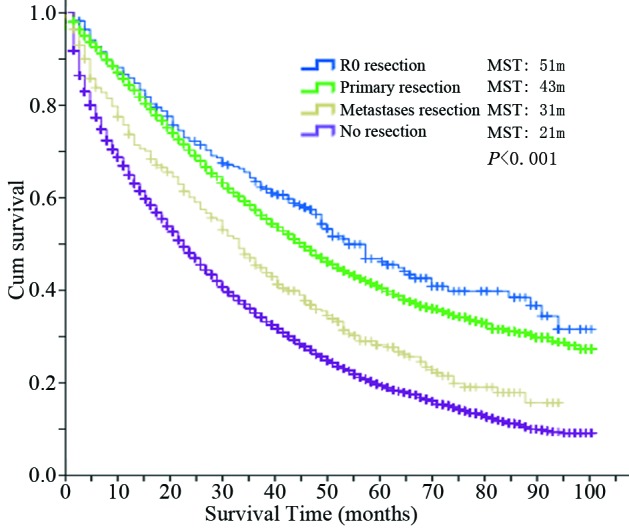
Overall survival curves of the four groups

According to the follow-up results, 7,125 events (breast cancer-specific deaths) were observed. From the Kaplan-Meier survival curves for these four groups, which are shown in Figure 1, the R0 group showed the best overall survival outcome with a median survival time (MST) of 51 months, followed by the primary resection group (MST = 43 months) and metastases resection group (MST = 31 months). The no resection group achieved an MST of 21 months. The difference among the four groups was significant (*P* < 0.001).

### Subgroup analysis of overall survival

The stratified analyses based on age at diagnosis, race, grade, tumor stage, radiation, HR status and metastatic site were conducted to explore the differences among the four groups and to determine which subgroup would benefit most from surgery. The survival results of each subgroup are shown in Table 2. It is obvious that the three groups with surgery achieved better MST than the no resection group in each subgroup.

**Table 2:** Stratified analyses results of median survival time

Variance	R0 resection	Primary resection	Metastases resection	No resection	Total	*P*value^a^
Age						
≤45years	57	51	49	26	39	<0.001
>45years	51	41	29	20	28	<0.001
Race						
White	54	44	31	22	31	<0.001
Black	25	30	26	15	20	<0.001
Other	84	58	64	24	40	<0.001
Grade						
Well	NA	70	50	33	45	<0.001
Moderate	66	58	34	28	41	<0.001
Poor	35	34	22	18	26	<0.001
Stage T						
T0	34	34	NA	24	30	0.006
T1	54	62	38	25	42	<0.001
T2	66	52	33	27	40	<0.001
T3	NA	37	45	26	34	<0.001
T4	33	32	16	18	23	<0.001
Radiation						
Done	54	47	31	20	33	<0.001
None	50	39	31	22	27	<0.001
HR						
HR+	66	52	38	28	38	<0.001
HR-	18	24	12	12	17	<0.001
Metastatic site						
Distant lymph node	NA	NA	33	27	62	<0.001
Designated organsb	46	42	32	23	31	<0.001
Other organs	48	43	31	20	28	<0.001
Multiplec	38	26	18	17	21	<0.001
Total	51	43	31	21	29	<0.001

NA, not available.

a Comparison among the four groups.

b Designated organs, metastasis in the following organs: adrenal (suprarenal) gland, bone, other than the adjacent rib, contralateral (opposite) breast, lung, ovary, satellite nodule(s) in skin other than the primary breast.

c Multiple mean metastases in at least two of the above sites

### Multivariate analysis using the Cox proportional hazards model

The results of the multivariate analysis using the Cox proportional hazards model are shown in Table 3. Age over 45 years, black race, poor grade, T3/T4 stage, metastases in organs and HR- status were independently associated with decreased OS, while other race and the three surgery groups were independently associated with increased OS. Compared with the no resection group, the R0 group showed the best hazard ratio of 0.558 (95% CI, 0.471-0.661), the primary resection group and metastases resection group also had a good survival benefit with a hazard ratio of 0.590 (95% CI, 0.557-0.625) and 0.782 (95% CI, 0.693-0.883).

**Table 3:** Results of multivariate analysis using the Cox proportional hazards model

Variance	*P* value	Hazard ratio	95.0% Confidence interval
**Age**			
≤45years		1	
>45years	<0.001	1.320	1.231-1.416
**Race**			
White		1	
Black	<0.001	1.280	1.204-1.362
Other	<0.001	0.797	0.719-0.882
**Grade**			
Well		1	
Moderate	0.131	1.097	0.973-1.237
Poor	<0.001	1.440	1.278-1.622
**Stage T**			
T1^a^		1	
T2	0.341	1.046	0.954-1.147
T3	0.006	1.163	1.045-1.294
T4	<0.001	1.384	1.266-1.512
**Radiation**			
Done		1	
None	0.847	0.995	0.946-1.046
**HR**			
Positive		1	
Negative	<0.001	1.714	1.616-1.817
**Surgery**			
No resection		1	
R0 resection	<0.001	0.558	0.471-0.661
Primary resection	<0.001	0.590	0.557-0.625
Metastases resection	<0.001	0.782	0.693-0.883
**Metastatic site**			
Distant lymph node		1	
Designated organs^b^	<0.001	1.838	1.622-2.083
Other organs	<0.001	1.839	1.622-2.084
Multiple^c^	<0.001	2.245	1.962-2.565

a T0 merged into T1 when doing Cox multivariate analysis

b Designated organs, metastasis in the following organs: adrenal (suprarenal) gland, bone, other than the adjacent rib, contralateral (opposite) breast, lung, ovary, satellite nodule(s) in skin other than the primary breast.

c Multiple mean metastases in at least two of the above sites.

### Different HR status shows a different survival benefit from surgery

**Figure 2:**
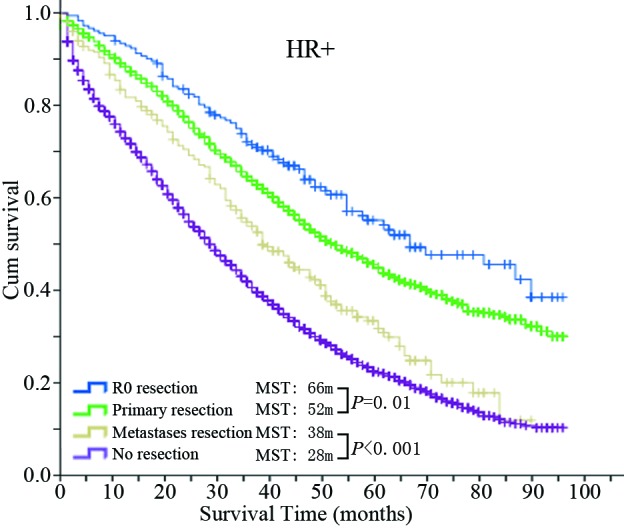
Kaplan-Meier survival curves of the four groups in the HR+ population

The MST and 5-year OS of the four groups based on different HR status are shown in Table 4. The corresponding Kaplan-Meier survival curves are shown in Figure 2. In the HR+ population, the survival of each group with surgery, including the R0 resection group (MST = 66 m; 5-year OS = 54.1%), primary resection group (MST = 52 m; 5-year OS = 44.9%) and metastases resection group (MST = 38 m; 5-year OS = 31.7%) were all significantly longer than the no resection group (MST = 28 m; 5-year OS = 22.0%) (*P* values were all < 0.001). Furthermore, the R0 group gained an additional survival benefit compared with the primary resection group (*P* value <0.001). On the other hand, in the HR- population, the survivals of the R0 group (MST = 18 m; 5-year OS = 26.7%) and primary resection group (MST = 24 m; 5-year OS = 25.0%) were both significantly longer than the no resection group (MST = 12 m; 5-year OS = 11.8%) but not the metastases resection group (MST = 12 m, 5-year OS = 6.8%) (*P* = 0.526). Furthermore, the R0 group did not achieve a better survival benefit compared with the primary resection group (*P* = 0.691).

### Univariate and multivariate logistic regression as predictors for surgery

To assess the interaction of HR positivity and the role of surgery, three surgery groups were merged into one group, the surgery group. Univariate logistic regression analysis (results are shown in Supplementary Table 2) by HR positivity revealed that HR-negative patients were significantly more likely to undergo surgery (OR = 1.201; *P* < 0.001); however, after adjusting for other factors, multivariate analysis (results are shown in Supplement Table 3) showed that HR positivity was not independently associated with greater or less likelihood of surgery (*P* = 0.814).

**Table 4:** Median survival time and 5-year overall survival of the four groups based on different hormone receptor status

HR+					HR-			
	MST	5-year OS	*P*value^*^	*P* value#	MST	5-year OS	*P* value^*^	*P*value#
R0 resection	66 m	54.1%	<0.001	0.011	18 m	26.7%	0.002	0.691
Primary resection	52 m	44.9%	<0.001		24 m	25.0%	<0.001	
Metastases resection	38 m	31.7%	<0.001	<0.001	12 m	6.8%	0.526	<0.001
No resection	28 m	22.0%		<0.001	12 m	11.8%		<0.001

* Compared with the no resection group;

# compared with the primary resection group; ER, estrogen receptor; PR, progesterone receptor; HR+, ER+ or PR+; HR-, both ER- and PR-. OS, overall survival rate.

**Figure 3:**
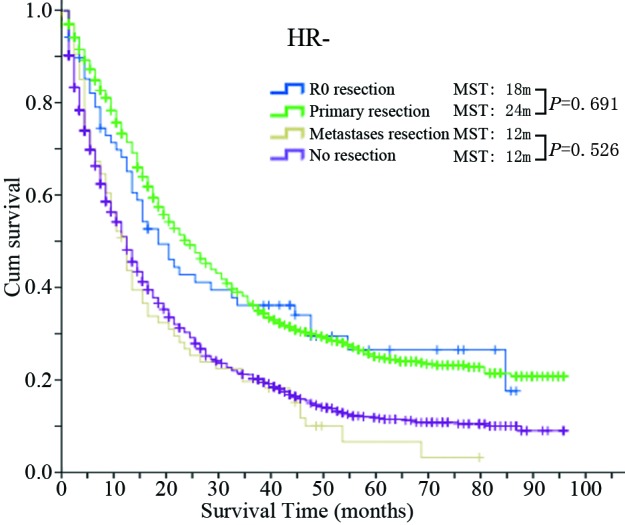
Kaplan-Meier survival curves of the four groups in the HR- population

## DISCUSSION

The present study showed that a survival gain was observed in operated stage IV breast cancer compared with the no resection group. After adjusting for other confounders, multivariate analysis showed that the three surgery groups were independently associated with increased OS. In the HR+ population, survival was best in the R0 group, and metastases resection somehow gained a survival benefit; however, in the HR- population, no significant benefit was attained in the R0 group compared with the primary resection group; additionally, no significant benefit was achieved in the metastases resection group compared with the no resection group.

Recent advances in chemotherapy, endocrine therapy and targeted therapy have achieved a rapid response and increased survival in most metastatic breast cancer patients[2]; thus, the standard treatment for stage IV breast cancer patients remains medical treatment, and the role of surgery in stage IV breast cancer patients is still controversial[3]. Several positive survival outcomes after surgery, with or without chemotherapy/endocrine therapy, were associated with a long disease-free interval after treatment of the primary tumor (12-36 months),[4–14] complete resection of the tumor,[15, 16] and ER+ status.[6] Additionally, a meta-analysis of 15 studies demonstrated that surgery of the primary tumor was independently associated with improved survival.[17] There were also some retrospective studies that found surgery to be associated with improved survival in patients with ER/PR-positive disease, while little or no survival benefit was observed in those with triple-negative disease.[7, 13] Some people may argue that the survival benefit associated with surgery in these retrospective studies may be due to the selection biases.[18] Therefore, several Phase III randomized controlled trials are being performed to determine whether local therapy would prolong survival in stage IV breast cancer patients.[1, 19] In the study from Tata Memorial Hospital, the median overall survival was 19.2 months in the locoregional treatment group and 20.5 months in the no locoregional treatment group (Hazad ratio = 1.04; *P* = 0.79), and no subgroup showed a significant survival benefit from surgical excision, including the estrogen receptor/progesterone receptor-positive and -negative groups.[20] Another phase III study from the Turkish Federation of Societies for Breast Disease also demonstrated that the locoregional treatment and no locoregional treatment groups had similar overall survival rates (35% in the surgery group and 31% in the no surgery group). In our retrospective study, selection biases may somehow play a role in the survival benefit of these four group comparisons; however, the selection bias may be less between the HR+ population and HR- population. The survival difference between the R0 resection group and primary resection groups in the HR+ population was significant, but not in the HR- population. Additional survival benefit from metastatic site resection was gained in the HR+ population compared with the HR- population. Similarly, the metastases resection group and no resection group showed different survival outcomes in the HR+ population compared with the HR- population. These re sults may not directly provide evidence to suggest surgical treatment in metastatic breast cancer but may inspire us to explore a more aggressive approach for HR+ breast cancer patients, in whom a survival benefit may be more prone to appear, compared with the HR- population.

The SEER program provided access to a large cohort of patients, making the study results more reliable. However, several limitations remain in our study. First, the information on the patient status was not accessible. There are several important factors associated with the survival of stage IV breast cancer patients, including performance status, number of metastatic sites, HER2 status, endocrine therapy, and chemotherapy. Particularly, the size of the metastases would determine whether it is resectable. Although the SEER program did not include this information, our present study could show, to a certain extent, that cytoreductive surgery could provide a survival benefit in HR+ stage IV breast cancer.

## MATERIALS AND METHODS

### Data source

The SEER program included 17 population-based cancer registries, together comprising approximately 28% of the total population of the United States.[21–24] Women initially diagnosed with stage IV primary invasive breast cancer from the 2004 to 2008 year were included in the analysis. Patients after year 2008 were not included to ensure a sufficiently long follow-up time.

### Patient selection

Breast cancer patient data were obtained from the SEER Program using Case Listing. Site record ICD-O-3 was limited to the breast. Case patients with sarcomas of the breast (based on the histology codes 8800, 8801, 8805, 8815, 8830, 8850, 8858, 8890, 8935, 8980, 8982, 8983, 9120, 9180, 9181, and 9260) were excluded. Multiple primary cancers were also excluded to make accessible analyses of cancer-specific survival. The patients with a survival of less than one month were excluded because such patients may die of surgical complications.

All of the study data—including demographic characteristics, ER, PR status, tumor stage, grade, classification of metastatic site, surgery of primary site and distant site, radiotherapy, cause of death and survival months—were all collected. HER2 status was not collected because it was not available for the cases before year 2010.

### Variables of interest

Cancer-specific survival was calculated from the date of diagnosis to the date of death related to breast cancer. Death attributed to other causes was considered as censored observation. Cases of grade 4 in histology were combined with cases of grade 3 because the outcome for cases assigned grade 3 or grade 4 was not significantly different.[25] As recorded in the SEER database, the metastatic sites of breast cancer were classified into several groups as follows: 1. distant lymph node(s), 2. metastases in designated organs: adrenal (suprarenal) gland, bone, other than adjacent rib, contralateral (opposite) breast, lung, ovary, satellite nodule(s) in skin other than primary breast, 3. metastases in other organs, 4. metastases in at least two of the above sites, 5. unknown.

The ER and PR results were combined and analyzed jointly as hormone receptor (HR) status. HR+ was defined as ER+ or PR+. HR- was defined as both ER- and PR-. ER/PR borderline case patients (borderline ER: *n* = 22, 0.2%; borderline PR: *n* = 78, 0.7%) were defined as unknown. Age at diagnosis was categorized as ≤ 45 years old versus >45 years old. All patients were divided into the following four groups: R0 group (patients who underwent primary site resection and distant metastatic site resection); primary resection group (patients who underwent only primary site resection); metastases resection group (patients who underwent only distant metastatic site resection); and no resection group (no resection was performed on any patient).

### Statistical analysis

The observed differences between the different surgery groups were analyzed statistically by chi-squared test. Univariate analysis of survival was performed using Kaplan-Meier methods, while group comparisons were performed using the log-rank test. Adjusted hazard ratios along with 95% intervals were calculated using the Cox regression model. Additionally, univariate and multivariate logistic regression analyses were applied to assess factors, especially hormone receptor status, associated with undergoing surgery. Differences were considered to be statistically significant if the *P* value was less than 0.05. SPSS 19.0 software (IBM Corp., Armonk, USA) was used for data analysis.

## SUPPLEMENTARY FIGURES AND TABLES








