# Supplementary material for: Hormone receptor status may impact the survival benefit of surgery in stage IV breast cancer: a population-based study
Source: Oncotarget. 2016 Aug 11;7(43):70991–1000. doi: 10.18632/oncotarget.11235 (PMC5342604; doi:10.18632/oncotarget.11235)
Supplement: Supplementary file 1 [file oncotarget-07-70991-s001.pdf]

Hormone receptor status may impact the survival benefit of surgery in stage iv breast cancer: a population-based study

Supplementary Material

Supplement Table 1: Clinical and pathological features of the study population

Variance	HR+ (n=6554)	HR- (n=2401)	Unknown (n=1486)	*P* value*
**Age (%)**				<0.001
≤45years	917 (14.0)	447 (18.6)	136 (9.2)	
>45years	5637 (86.0)	1954 (81.4)	1350 (90.8)	
**Race (%)**				<0.001
White	5181 (79.1)	1692 (70.5)	1132 (76.2)	
Black	907 (13.8)	529 (22.0)	272 (18.3)	
Other	444 (6.8)	174 (7.2)	74 (5.0)	
Unknown	22 (0.3)	6 (0.2)	8 (0.5)	
**Grade (%)**				<0.001
Well	515 (7.9)	37 (1.5)	32 (2.2)	
Moderate	2356 (35.9)	394 (16.4)	186 (12.5)	
Poor	2286 (34.9)	1607 (66.9)	337 (22.7)	
Unknown	1397 (21.3)	363 (15.1)	931 (62.7)	
**Stage T (%)**				<0.001
T0	127 (1.9)	25 (1.0)	54 (3.6)	
T1	794 (12.1)	267 (11.1)	85 (5.7)	
T2	1752 (26.7)	530 (22.1)	203 (13.7)	
T3	757 (11.6)	303 (12.6)	80 (5.4)	
T4	2039 (31.1)	948 (39.5)	381 (25.6)	
Tx	1085 (16.6)	328 (13.7)	683 (46.0)	
**Stage N (%)**				<0.001
0	1485 (22.7)	444 (18.5)	285 (19.2)	
1	2224 (33.9)	865 (36.0)	371 (25.0)	
2	800 (12.2)	282 (11.7)	82 (5.5)	
3	897 (13.7)	477 (19.9)	93 (6.3)	
NX	1148 (17.5)	333 (13.9)	655 (44.1)	
**Radiation (%)**				0.033
Done	2484 (37.9)	847 (35.3)	355 (23.9)	
None	3950 (60.3)	1498 (62.4)	1093 (73.6)	
Unknown	120 (1.8)	56 (2.3)	38 (2.6)	
**Surgery (%)**				<0.001
R0 resection	184 (2.8)	70 (2.9)	18 (1.2)	
Primary resection	2646 (40.4)	1095 (45.6)	284 (19.1)	
Metastases resection	252 (3.8)	74 (3.1)	83 (5.6)	
No resection	3472 (53.0)	1162 (48.4)	1101 (74.1)	
**Metastatic site (%)**				<0.001

Distant lymph node	324 (4.9)	212 (8.8)	60 (4.0)
Designated organs	3003 (45.8)	798 (33.2)	562 (37.8)
Other organs	2316 (35.3)	935 (38.9)	662 (44.5)
Multiple	837 (12.8)	412 (17.2)	162 (10.9)
Unknown	74 (1.1)	44 (1.8)	40 (2.7)

HR+ was defined as ER+ or PR+. HR- was defined as both ER- and PR-.

* Comparison between HR+ and HR- populations.

Supplement Table 2: Univariate analyses of factors predicting surgery[a]

**Variance**	**Sample size**	**% of sample size**	**Surgery rate**	**OR**	*P* value[b]
**Age**					
≤45years	1500	14.4%	55.7%	Ref	
>45years	8941	85.6%	43.3%	0.606	<0.001
**Race**					
White	8005	76.7%	45.5%	Ref	
Black	1708	16.4%	42.1%	0.871	0.010
Other	692	6.6%	48.1%	1.111	0.184
Unknown	36	0.3%	33.3%	0.599	0.148
**Grade**					
Well	584	5.6%	51.4%	Ref	
Moderate	2936	28.1%	50.3%	0.957	0.628
Poor	4230	40.5%	55.9%	1.198	0.041
Unknown	2691	25.8%	21.1%	0.253	<0.001
**Stage T**					
T1	1146	11.0%	62.0%	Ref	
T2	2485	23.8%	61.8%	0.992	0.916
T3	1140	10.9%	58.4%	0.863	0.084
T4	3368	32.3%	41.1%	0.428	<0.001
Tx	2302	22.0%	17.9%	0.133	<0.001
**Stage N**					
0	2214	21.2%	42.6%	Ref	
1	3460	33.1%	43.5%	1.036	0.516
2	1164	11.1%	70.2%	3.173	<0.001
3	1467	14.1%	65.8%	2.591	<0.001
NX	2136	20.5%	22.3%	0.388	<0.001
**Radiation**					
Done	3686	35.3%	54.3%	Ref	
None	6541	62.6%	39.2%	0.542	<0.001

Unknown	214	2.0%	66.8%	1.696	<0.001
**ER**					
Positive	6420	61.5%	47.0%	Ref	
Negative	2550	24.4%	51.3%	1.190	<0.001
Unknown	1471	14.1%	25.8%	0.393	<0.001
**PR**					
Positive	4884	46.8%	47.3%	Ref	
Negative	3907	37.4%	50.0%	1.115	0.011
Unknown	1650	15.8%	26.8%	0.408	<0.001
**HR**					
HR+	6554	62.8%	47.0%	Ref	
HR-	2401	23.0%	51.6%	1.201	<0.001
Unknown	1486	14.2%	25.9%	0.394	<0.001
**Metastatic site**					
Distant lymph node	596	5.7%	67.8%	Ref	
Designated organs[c]	4363	41.8%	45.2%	0.391	<0.001
Other organs	3913	37.5%	44.7%	0.384	<0.001
Multiple[d]	1411	13.5%	35.3%	0.259	<0.001
Unknown	158	1.5%	53.8%	0.553	0.001

OR odds ratio. ER, estrogen receptor; PR, progesterone receptor; HR+ was defined as ER+ or PR+. HR- was defined as both ER- and PR-.

[a] Surgery including the R0 resection, primary resection and metastases resection groups.

[b] OR of surgery (vs. no surgery) and *P* value from univariate logistic regression

[c] Designated organs, metastasis in the following organs: adrenal (suprarenal) gland, bone, other than the adjacent rib, contralateral (opposite) breast, lung, ovary, satellite nodule(s) in skin other than the primary breast.

[d] Multiple mean metastases in at least two of the above sites.

Supplement Table 3: Multivariate model of factors predicting surgery

Variance	*P* value[a]	OR	95%CI
**Age**			
<=45years old		Ref	
>45years old	0.039	0.842	0.716-0.991
**Race**			
White		Ref	
Black	<0.001	0.736	0.628-0.863
Other	0.037	0.789	0.632-0.986
**Grade**			
Well		Ref	
Moderate	0.886	1.018	0.801-1.293
Poor	0.023	1.324	1.040-1.686
**Stage T**			
T1[a]		Ref	
T2	0.557	0.943	0.777-1.146
T3	<0.001	0.637	0.509-0.796
T4	<0.001	0.336	0.277-0.407
**Stage N**			
0		Ref	
1	0.158	1.114	0.959-1.295
2	<0.001	3.393	2.764-4.164
3	<0.001	3.159	2.601-3.837
**Radiation**			
Done		Ref	
None	<0.001	0.565	0.500-0.640
**HR**			
HR+		Ref	
HR-	0.814	0.983	0.856-1.130
**Metastatic site**			
Distant lymph node		Ref	
Designated organs[b]	<0.001	0.533	0.408-0.698
Other organs	<0.001	0.562	0.429-0.737
Multiple[c]	<0.001	0.244	0.182-0.328

OR, odds ratio. HR- was defined as both ER- and PR-.

[a] T0 merged into T1 when performing Cox multivariate analysis

[B] Designated organs, metastasis in the following organs: adrenal (suprarenal) gland, bone, other than the adjacent rib, contralateral (opposite) breast, lung, ovary, satellite nodule(s) in skin other than the primary breast.

[C] Multiple mean metastases in at least two of the above sites.
