# Supplementary material for: Hormone receptor status may impact the survival benefit of surgery in stage IV breast cancer: a population-based study
Source: Oncotarget. 2016 Aug 11;7(43):70991–1000. doi: 10.18632/oncotarget.11235 (PMC5342604; doi:10.18632/oncotarget.11235)
Supplement: Supplementary file 2 [file oncotarget-07-70991-s002.docx]

Supplement table 1 Clinical and pathological features of the study population

| **Variance** | **HR+ (n=6554)** | **HR- (n=2401)** | **Unknown (n=1486)** | ***P* value^*^** |
| --- | --- | --- | --- | --- |
| **Age (%)** |  |  |  | <0.001 |
| ≤45years | 917 (14.0) | 447 (18.6) | 136 (9.2) |  |
| >45years | 5637 (86.0) | 1954 (81.4) | 1350 (90.8) |  |
| **Race (%)** |  |  |  | <0.001 |
| White | 5181 (79.1) | 1692 (70.5) | 1132 (76.2) |  |
| Black | 907 (13.8) | 529 (22.0) | 272 (18.3) |  |
| Other | 444 (6.8) | 174 (7.2) | 74 (5.0) |  |
| Unknown | 22 (0.3) | 6 (0.2) | 8 (0.5) |  |
| **Grade (%)** |  |  |  | <0.001 |
| Well | 515 (7.9) | 37 (1.5) | 32 (2.2) |  |
| Moderate | 2356 (35.9) | 394 (16.4) | 186 (12.5) |  |
| Poor | 2286 (34.9) | 1607 (66.9) | 337 (22.7) |  |
| Unknown | 1397 (21.3) | 363 (15.1) | 931 (62.7) |  |
| **Stage T (%)** |  |  |  | <0.001 |
| T0 | 127 (1.9) | 25 (1.0) | 54 (3.6) |  |
| T1 | 794 (12.1) | 267 (11.1) | 85 (5.7) |  |
| T2 | 1752 (26.7) | 530 (22.1) | 203 (13.7) |  |
| T3 | 757 (11.6) | 303 (12.6) | 80 (5.4) |  |
| T4 | 2039 (31.1) | 948 (39.5) | 381 (25.6) |  |
| Tx | 1085 (16.6) | 328 (13.7) | 683 (46.0) |  |
| **Stage N (%)** |  |  |  | <0.001 |
| 0 | 1485 (22.7) | 444 (18.5) | 285 (19.2) |  |
| 1 | 2224 (33.9) | 865 (36.0) | 371 (25.0) |  |
| 2 | 800 (12.2) | 282 (11.7) | 82 (5.5) |  |
| 3 | 897 (13.7) | 477 (19.9) | 93 (6.3) |  |
| NX | 1148 (17.5) | 333 (13.9) | 655 (44.1) |  |
| **Radiation (%)** |  |  |  | 0.033 |
| Done | 2484 (37.9) | 847 (35.3) | 355 (23.9) |  |
| None | 3950 (60.3) | 1498 (62.4) | 1093 (73.6) |  |
| Unknown | 120 (1.8) | 56 (2.3) | 38 (2.6) |  |
| **Surgery (%)** |  |  |  | <0.001 |
| R0 resection | 184 (2.8) | 70 (2.9) | 18 (1.2) |  |
| Primary resection | 2646 (40.4) | 1095 (45.6) | 284 (19.1) |  |
| Metastases resection | 252 (3.8) | 74 (3.1) | 83 (5.6) |  |
| No resection | 3472 (53.0) | 1162 (48.4) | 1101 (74.1) |  |
| **Metastatic site (%)** |  |  |  | <0.001 |
| Distant lymph node | 324 (4.9) | 212 (8.8) | 60 (4.0) |  |
| Designated organs | 3003 (45.8) | 798 (33.2) | 562 (37.8) |  |
| Other organs | 2316 (35.3) | 935 (38.9) | 662 (44.5) |  |
| Multiple | 837 (12.8) | 412 (17.2) | 162 (10.9) |  |
| Unknown | 74 (1.1) | 44 (1.8) | 40 (2.7) |  |

HR+ was defined as ER+ or PR+. HR- was defined as both ER- and PR-.

* Comparison between HR+ and HR- populations.
