# Supplementary material for: Hormone receptor status may impact the survival benefit of surgery in stage IV breast cancer: a population-based study
Source: Oncotarget. 2016 Aug 11;7(43):70991–1000. doi: 10.18632/oncotarget.11235 (PMC5342604; doi:10.18632/oncotarget.11235)
Supplement: Supplementary file 3 [file oncotarget-07-70991-s003.docx]

Supplement table 2. Univariate analyses of factors predicting surgery^a^

| **Variance** | **Sample size** | **% of sample size** | **Surgery rate** | **OR** | *P* value^b^ |
| --- | --- | --- | --- | --- | --- |
| **Age** |  |  |  |  |  |
| ≤45years | 1500 | 14.4% | 55.7% | Ref |  |
| >45years | 8941 | 85.6% | 43.3% | 0.606 | <0.001 |
| **Race** |  |  |  |  |  |
| White | 8005 | 76.7% | 45.5% | Ref |  |
| Black | 1708 | 16.4% | 42.1% | 0.871 | 0.010 |
| Other | 692 | 6.6% | 48.1% | 1.111 | 0.184 |
| Unknown | 36 | 0.3% | 33.3% | 0.599 | 0.148 |
| **Grade** |  |  |  |  |  |
| Well | 584 | 5.6% | 51.4% | Ref |  |
| Moderate | 2936 | 28.1% | 50.3% | 0.957 | 0.628 |
| Poor | 4230 | 40.5% | 55.9% | 1.198 | 0.041 |
| Unknown | 2691 | 25.8% | 21.1% | 0.253 | <0.001 |
| **Stage T** |  |  |  |  |  |
| T1 | 1146 | 11.0% | 62.0% | Ref |  |
| T2 | 2485 | 23.8% | 61.8% | 0.992 | 0.916 |
| T3 | 1140 | 10.9% | 58.4% | 0.863 | 0.084 |
| T4 | 3368 | 32.3% | 41.1% | 0.428 | <0.001 |
| Tx | 2302 | 22.0% | 17.9% | 0.133 | <0.001 |
| **Stage N** |  |  |  |  |  |
| 0 | 2214 | 21.2% | 42.6% | Ref |  |
| 1 | 3460 | 33.1% | 43.5% | 1.036 | 0.516 |
| 2 | 1164 | 11.1% | 70.2% | 3.173 | <0.001 |
| 3 | 1467 | 14.1% | 65.8% | 2.591 | <0.001 |
| NX | 2136 | 20.5% | 22.3% | 0.388 | <0.001 |
| **Radiation** |  |  |  |  |  |
| Done | 3686 | 35.3% | 54.3% | Ref |  |
| None | 6541 | 62.6% | 39.2% | 0.542 | <0.001 |
| Unknown | 214 | 2.0% | 66.8% | 1.696 | <0.001 |
| **ER** |  |  |  |  |  |
| Positive | 6420 | 61.5% | 47.0% | Ref |  |
| Negative | 2550 | 24.4% | 51.3% | 1.190 | <0.001 |
| Unknown | 1471 | 14.1% | 25.8% | 0.393 | <0.001 |
| **PR** |  |  |  |  |  |
| Positive | 4884 | 46.8% | 47.3% | Ref |  |
| Negative | 3907 | 37.4% | 50.0% | 1.115 | 0.011 |
| Unknown | 1650 | 15.8% | 26.8% | 0.408 | <0.001 |
| **HR** |  |  |  |  |  |
| HR+ | 6554 | 62.8% | 47.0% | Ref |  |
| HR- | 2401 | 23.0% | 51.6% | 1.201 | <0.001 |
| Unknown | 1486 | 14.2% | 25.9% | 0.394 | <0.001 |
| **Metastatic site** |  |  |  |  |  |
| Distant lymph node | 596 | 5.7% | 67.8% | Ref |  |
| Designated organs^c^ | 4363 | 41.8% | 45.2% | 0.391 | <0.001 |
| Other organs | 3913 | 37.5% | 44.7% | 0.384 | <0.001 |
| Multiple^d^ | 1411 | 13.5% | 35.3% | 0.259 | <0.001 |
| Unknown | 158 | 1.5% | 53.8% | 0.553 | 0.001 |

OR odds ratio. ER, estrogen receptor; PR, progesterone receptor; HR+ was defined as ER+ or PR+. HR- was defined as both ER- and PR-.

^a^ Surgery including the R0 resection, primary resection and metastases resection groups.

^b^ OR of surgery (vs. no surgery) and *P* value from univariate logistic regression

^c^ Designated organs, metastasis in the following organs: adrenal (suprarenal) gland, bone, other than the adjacent rib, contralateral (opposite) breast, lung, ovary, satellite nodule(s) in skin other than the primary breast.

^d^ Multiple mean metastases in at least two of the above sites.
