# Supplementary material for: Hormone receptor status may impact the survival benefit of surgery in stage IV breast cancer: a population-based study
Source: Oncotarget. 2016 Aug 11;7(43):70991–1000. doi: 10.18632/oncotarget.11235 (PMC5342604; doi:10.18632/oncotarget.11235)
Supplement: Supplementary file 4 [file oncotarget-07-70991-s004.docx]

Supplement table 3 Multivariate model of factors predicting surgery

| **Variance** | ***P* value^a^** | **OR** | **95%CI** |
| --- | --- | --- | --- |
| **Age** |  |  |  |
| <=45years old |  | Ref |  |
| >45years old | 0.039 | 0.842 | 0.716-0.991 |
| **Race** |  |  |  |
| White |  | Ref |  |
| Black | <0.001 | 0.736 | 0.628-0.863 |
| Other | 0.037 | 0.789 | 0.632-0.986 |
| **Grade** |  |  |  |
| Well |  | Ref |  |
| Moderate | 0.886 | 1.018 | 0.801-1.293 |
| Poor | 0.023 | 1.324 | 1.040-1.686 |
| **Stage T** |  |  |  |
| T1^a^ |  | Ref |  |
| T2 | 0.557 | 0.943 | 0.777-1.146 |
| T3 | <0.001 | 0.637 | 0.509-0.796 |
| T4 | <0.001 | 0.336 | 0.277-0.407 |
| **Stage N** |  |  |  |
| 0 |  | Ref |  |
| 1 | 0.158 | 1.114 | 0.959-1.295 |
| 2 | <0.001 | 3.393 | 2.764-4.164 |
| 3 | <0.001 | 3.159 | 2.601-3.837 |
| **Radiation** |  |  |  |
| Done |  | Ref |  |
| None | <0.001 | 0.565 | 0.500-0.640 |
| **HR** |  |  |  |
| HR+ |  | Ref |  |
| HR- | 0.814 | 0.983 | 0.856-1.130 |
| **Metastatic site** |  |  |  |
| Distant lymph node |  | Ref |  |
| Designated organs^b^ | <0.001 | 0.533 | 0.408-0.698 |
| Other organs | <0.001 | 0.562 | 0.429-0.737 |
| Multiple^c^ | <0.001 | 0.244 | 0.182-0.328 |

OR, odds ratio. HR- was defined as both ER- and PR-.

^a^ T0 merged into T1 when performing Cox multivariate analysis

^B^ Designated organs, metastasis in the following organs: adrenal (suprarenal) gland, bone, other than the adjacent rib, contralateral (opposite) breast, lung, ovary, satellite nodule(s) in skin other than the primary breast.

^C^ Multiple mean metastases in at least two of the above sites.
